# Spatially resolved EEG reveals theta-band network modulation following iTBS in aging and mild cognitive impairment

**DOI:** 10.3389/fnhum.2026.1741133

**Published:** 2026-03-26

**Authors:** Lawrence R. Frank, Vitaly L. Galinsky, Hangbin Zhang, John D. Hall, Mark W. Bondi, Ying-hui Chou

**Affiliations:** 1 Center for Scientific Computation in Imaging, University of California at San Diego, La Jolla, CA, United States; 2 Center for Functional MRI, University of California at San Diego, La Jolla, CA, United States; 3 Brain Imaging and Transcranial Magnetic Stimulation Laboratory, University of Arizona, Tucson, AZ, United States; 4 Department of Psychiatry, University of California at San Diego, La Jolla, CA, United States

**Keywords:** brain physics, brain waves, brain networks, electroencephalography, neuroimaging, transcranial magnetic stimulation (TMS), mild cognitive impairment (MCI)

## Abstract

Neuromodulation shows promise as a general strategy for non-pharmacological intervention in a range of psychiatric and neurodegenerative brain disorders. Two major challenges in making neuromodulation methods clinically viable are (1) Assessing brain network changes induced by the stimulation, and (2) Optimizing stimulation protocols by adjusting the locations and spectral content of the stimulation. Spatially resolved electroencephalography (EEG) provides solutions to both by characterizing whole brain frequency-dependent *brain electrical networks* (BENs) with high spatial and temporal resolution. In this paper, we describe our novel *SPatially resolved EEG Constrained with Tissue properties by Regularized Entropy* (SPECTRE) method, the first to reconstruct whole-brain, high spatially and temporally resolved brain electrical networks (BENs) from standard EEG data. We apply SPECTRE to a sham-controlled transcranial magnetic stimulation (TMS) intervention in older adults spanning cognitively normal (CN) aging and mild cognitive impairment (MCI). In this pilot case series, SPECTRE detected consistent theta-band BEN changes associated with active intermittent theta-burst stimulation(iTBS) relative to sham, suggesting the potential for TMS to modulate affected deep brain regions in older adults. These preliminary results warrant replication in larger samples. This study further suggests that accelerated iTBS might function by targeting compensatory networks in MCI. SPECTRE Overall, the present results support the feasibility of using SPECTRE-enhanced EEG to track stimulation-associated network changes and motivate future work testing whether such measures can inform individualized neuromodulation protocols.

## 1 Introduction

Neuromodulation is a promising non-pharmacological approach for psychiatric and neurodegenerative disorders. Among available methods, *Transcranial Magnetic Stimulation* (TMS) modulates *brain electrical networks* (BENs). Repetitive TMS (rTMS) is widely used as a non-invasive stimulation technique capable of altering brain activity and network organization beyond the stimulation period (Klomjai et al., 2015; Lefaucheur et al., 2020). Currently FDA-approved for depression (Kazemi et al., 2025), rTMS is also being explored for Alzheimer's disease (AD) and Mild Cognitive Impairment (MCI) (Chou et al., 2020).

Although rTMS has been increasingly explored in AD and MCI, the clinical efficacy literature remains heterogeneous and modest. Recent reviews and meta-analyses report small and variable effect sizes, with outcomes strongly dependent on stimulation parameters, cortical targets, cognitive domain assessed, and disease stage (Chou et al., 2020; Zhang et al., 2025). Across studies, reported benefits are often domain-specific, inconsistent across time, or indistinguishable from sham stimulation. These mixed findings indicate that, despite theoretical motivation, the mechanisms by which TMS engages disease-relevant brain networks in aging populations remain insufficiently characterized, warranting mechanism-based investigation to improve efficacy.

Accelerated intermittent Theta Burst Stimulation (iTBS) schedules have been shown to produce clinical effects comparable to, and in some cases greater than, standard once-daily protocols in recent reviews (Cole et al., 2024; Zhang et al., 2025). Such schedules may be advantageous for probing stimulation-induced changes in memory-related networks in MCI.

An increasingly common strategy is to combine TMS with neuroimaging, most often Magnetic Resonance Imaging (MRI), to guide target selection and individualize stimulation protocols. Emerging evidence suggests that such individualized approaches yield more aligned and consistent response outcomes, indicating potential clinical progress of TMS through improved engagement of individualized brain networks (Jung et al., 2024; Koch et al., 2024). However, MRI-guided approaches are limited by high cost, restricted temporal resolution, and reduced accessibility to repeated monitoring and evaluation, which hinders their clinical adoption. Moreover, these expenses constrain widespread implementation, independent verification, and further generalization to broader populations. These limitations have motivated the exploration of electroencephalography (EEG) as a complementary or alternative approach.

EEG offers excellent temporal resolution and sensitivity to large-scale network dynamics. Resting-state biomarkers, including spectral power, connectivity measures, and event-related synchronization or desynchronization, have been linked to cognitive decline, including alterations in theta and alpha oscillations associated with memory and executive control (Duff, 2012; Park and Reuter-Lorenz, 2009; Reinhart and Nguyen, 2019). Nevertheless, conventional EEG suffers from poor spatial localization relative to MRI, limiting its ability to precisely map distributed network alterations in aging brains.

Despite its promise, two major barriers limit the clinical impact of neuromodulation. First, direct and concurrent measures of stimulation-induced network changes remain scarce. Without such measures, it is difficult to confirm underlying mechanisms or monitor progression across repeated sessions. Standard neuropsychological tests, while widely used, are only indirect proxies of brain function. They are confounded by environment, expectation, and reporting bias, and cannot be administered during stimulation (Duff, 2012). As a result, they provide limited mechanistic insight and are unsuitable for guiding real-time adjustments, highlighting the critical role of neuroimaging. Second, optimizing stimulation protocols requires tools that can localize and quantify network responses with high spatiotemporal precision. Current methods force a trade-off: MRI offers good spatial resolution but poor temporal resolution, while EEG provides excellent temporal resolution but poor spatial localization. This trade-off constrains our ability to track stimulation effects in real time and hinders the development of individualized, adaptive protocols.

These barriers underscore the need for advanced methods that can deliver spatially and temporally precise measurements of stimulation-induced brain network changes. Overcoming these limitations would not only accelerate clinical translation but also deepen mechanistic understanding of neuromodulation.

To overcome the trade-off between spatial and temporal resolution, we developed the *SPatially resolved EEG Constrained with Tissue properties by Regularized Entropy* (SPECTRE) framework (Frank et al., 2025). Contrary to the long-standing belief that scalp EEG cannot resolve distributed electric fields, SPECTRE applies a Bayesian field-theoretic algorithm that reconstructs voxel-wise, frequency-resolved BENs. By incorporating tissue geometry and spatial correlations, it produces probabilistic maps of whole-brain activity with high spatiotemporal precision. Validation across simultaneous EEG/fMRI, intracranial EEG, and benchmark tasks demonstrates that SPECTRE matches, or in some cases exceeds, the spatial accuracy of fMRI while retaining EEG's hallmark millisecond resolution (Frank et al., 2025).

Unlike MRI, which is constrained by slow hemodynamic coupling and susceptibility-related distortions, SPECTRE-EEG reconstructs neural activity directly from electric fields, providing both high temporal precision and spatial fidelity. This dual advantage positions SPECTRE as a uniquely powerful imaging method for applications where precise mapping of brain dynamics is essential.

In particular, SPECTRE enables real-time detection of whole-brain networks engaged by neuromodulation, opening the door to optimized, clinically viable, and patient-specific protocols. These capabilities are especially important for conditions such as Alzheimer's Disease and related dementias (ADRD), where enhancing or restoring memory-related networks may depend on tailoring interventions to the dynamic state of each patient's brain.

These advances directly address critical barriers in neuromodulation. Progress depends on the ability to (1) localize stimulation targets precisely, (2) monitor frequency-specific BEN dynamics with spatial fidelity, and (3) quantify stimulation-induced changes in real time to enable individualized, adaptive protocols. By transforming EEG into a modality that provides both spatial and temporal precision in an accessible and scalable format, SPECTRE establishes the methodological foundation needed to enhance the clinical impact of TMS.

Here, we present the first application of SPECTRE in a rigorously characterized case series of older adults, combining high-density EEG, accelerated iTBS, and detailed cognitive profiling to test whether repeated stimulation sessions induce measurable changes in BENs. We hypothesize that SPECTRE-enhanced EEG will enable the detection and localization of frequency-specific, whole-brain changes in BENs induced by iTBS in older adults, including those with MCI, in this pilot sample. In particular, we expect alterations in theta-band networks implicated in executive functioning that differ from those observed under sham stimulation.

## 2 Methods

### 2.1 Subjects

In this case-series pilot study, eleven older adults (4 males, 7 females; mean age = 68.09 ± 6.64 years; mean education = 18.0 ± 2.10 years) with subjective cognitive complaints were recruited from the community. None of the participants reported a prior clinical diagnosis of cognitive impairment, Alzheimer's disease, or other neuropsychiatric disorders.

Based on the Jak/Bondi actuarial criteria applied to the Uniform Data Set version 3 (UDS-3) battery (Jak et al., 2009; Bondi et al., 2014), 4 participants were classified as cognitively normal and seven as having mild cognitive impairment (MCI). Global cognition was additionally assessed using the Montreal Cognitive Assessment (MoCA; mean = 27.27 ± 1.56). Neuropsychological results, reported as normative Z-scores, are presented in Table 1. Clinical review of MRI scans revealed no evidence of overt brain atrophy or neurological impairment. Written informed consent was obtained from all participants. All procedures were approved by the University of Arizona IRB and adhered to current TMS safety guidelines.

**Table 1:** Profile of baseline neuropsychological measurements of the Uniform Data Set version 3 (UDS3) neuropsychological battery and the Rey Auditory Verbal Learning Test (AVLT), adjusted for age, sex, and education (z-scores).

**Domains**	**Task**	MCI^*^(***n*** = **7)**		**HC (*****n*** = **4)**		**Total (*****n*** = **11)**	
		**Mean**	**SD**	**Mean**	**SD**	**Mean**	**SD**
General	MoCA total score	−0.38	0.60	0.79	0.32	0.04	0.77
Memory/visuospatial	Immediate craft story recall (verbatim scoring)	−0.60	0.84	−0.65	1.36	−0.62	0.99
	Immediate craft story recall (paraphrase scoring)	−0.75	0.75	−0.86	1.28	−0.79	0.91
	Delayed craft story recall (verbatim scoring)	−0.63	0.84	0.00	0.85	−0.40	0.86
	Delayed craft story recall (paraphrase scoring)	−1.06	0.74	−0.31	1.02	−0.79	0.89
	Total score for copy of Benson figure	0.04	0.19	−0.59	0.57	−0.19	0.47
	Total score for delayed drawing of Benson figure	−0.25	0.95	−0.06	0.22	−0.18	0.75
	AVLT: Learning Efficiency Sum	−0.30	0.77	0.47	0.88	−0.02	0.86
	AVLT: Delayed Recall Sum	0.06	1.38	0.77	1.26	0.31	1.32
	AVLT: Percent Retention Sum	0.02	1.40	0.87	1.04	0.33	1.30
Attention/working memory	Forward number span test (correct trials)	0.05	0.38	0.07	0.30	0.06	0.34
	Forward number span test (longest span)	−0.24	1.51	0.15	1.02	−0.10	1.31
	Backward number span test (correct trials)	−0.07	1.38	0.17	0.83	0.02	1.17
	Backward number span test (longest span)	−0.15	1.54	0.20	0.95	−0.02	1.31
Language	Multilingual naming test (MINT)	−0.06	0.62	0.16	0.82	0.02	0.67
	Number of correct F-words generated	−0.60	0.91	−0.06	0.83	−0.40	0.88
	Number of correct L-words generated	−0.28	0.61	1.06	0.94	0.21	0.97
	Number of correct F-words and L-words	−0.22	0.61	0.60	0.70	0.08	0.74
	Category fluency (animals)	−0.48	1.43	0.95	1.25	0.04	1.49
	Category fluency (vegetables)	−0.46	1.48	0.85	0.71	0.02	1.38
Attention/executive	Trail making test Part A	−1.04	0.90	0.19	0.89	−0.60	1.05
	Trail making test Part B	−0.75	0.61	0.81	1.17	−0.18	1.12

AVLT, The Rey Auditory-Verbal Learning Test; MoCA, The Montreal Cognitive Assessment.

^*^MCI group composed of 4 amnestic MCI and 3 non-amnestic MCI participants.

### 2.2 Experimental design

This study employed a randomized, double-blind, counterbalanced crossover design. Each participant completed both active and sham iTBS conditions, separated by a minimum one-month washout interval. On each experimental visit, participants received three stimulation sessions separated by 50-min rest intervals. Prior evidence indicates that single-day iTBS induces transient increases in cortical excitability that typically return toward baseline within approximately 1 h after stimulation (Alix et al., 2019; Yu et al., 2020), and crossover TMS/iTBS studies have commonly employed washout intervals of 1 week to one month to mitigate carryover effects (Nauczyciel et al., 2014; Teferi et al., 2024). The one-month interval used here is therefore conservative relative to prior designs and was selected to minimize the potential influence of residual stimulation effects across visits.

### 2.3 iTBS protocol and stimulation parameters

Stimulation was delivered with a figure-of-eight coil (MagPro X100, MagVenture, Denmark) and guided by stereotaxic infrared neuronavigation (Localite TMS Navigator 3D, Germany). The iTBS protocol consisted of bursts of three 50 Hz pulses repeated at 5 Hz (2 s trains, 10 s inter-train interval), producing 600 pulses per session (1,800 per day).

Individualized stimulation targets were identified using diffusion tensor imaging (DTI) tractography to locate parietal regions structurally connected to the left hippocampus (Chen et al., 2022). This approach is motivated by evidence that episodic memory depends on interactions between the hippocampus and distributed neocortical networks, including posterior parietal cortex (Barnett et al., 2021). Prior work has demonstrated that frequency-specific TBS delivered to connectivity-defined parietal sites can elicit distinct hippocampal activity patterns and selectively modulate hippocampal theta oscillations, with effect magnitude scaling with individual connectivity strength (Hermiller et al., 2019; Li et al., 2025). In addition, repetitive stimulation of parietal cortex targets defined by hippocampal connectivity has been associated with increased hippocampal-network functional connectivity and correlated improvements in memory behavior (Freedberg et al., 2019). By leveraging subject-specific white-matter pathways, tractography-based targeting increases the specificity of stimulation, enabling more precise engagement of memory-relevant networks than traditional anatomical landmark-based or purely functional approaches (Liu et al., 2024).

Targets were implemented using a neuronavigation system that provided real-time feedback to maintain coil orientation and positional accuracy. Resting motor threshold (RMT) was estimated using the TMS Motor Threshold Assessment Tool 2.0 (https://www.clinicalresearcher.org/software.htm), which uses a maximum-likelihood parameter estimation strategy with sequential testing and does not require a priori information (Borckardt et al., 2006). Stimulation intensity for iTBS was set at 80% of each participant's RMT. Across participants, the mean stimulator output corresponding to 80% RMT was 38.00% (*SD* = 6.48%) of maximum stimulator output. For the sham condition, a MagVenture sham TBS coil was used; it is identical in appearance, weight, and sound to the active coil but contains an internal magnetic absorption plate that attenuates the magnetic field and prevents cortical stimulation. Sham procedures mirrored the active condition, including coil positioning and neuronavigation setup. All sessions were administered by the same TMS research specialist, who was blinded to condition.

### 2.4 EEG acquisition and pre-processing

Resting-state EEG was recorded for 6 min immediately before the first stimulation session and again following the final session of each stimulation day (three iTBS sessions) using a 32-channel, TMS-compatible ActiChamp Plus system (Brain Products, Munich, Germany).

Electrodes were placed according to the international 10–20 system, with FCz as reference and AFz as ground. Signals were digitized at 1 kHz, with impedance maintained below 5*kΩ*. Participants were instructed to remain relaxed and fixate on a central crosshair.

EEG was recorded using a TMS-compatible cap that remained in place throughout the experimental session. Electrode impedances were checked regularly and conductive gel was reapplied as needed prior to each recording block. EEG acquisition began approximately 6 min after completion of iTBS.

Data were preprocessed as follows. Raw EEG was high-pass filtered at 0.5 Hz, low-pass filtered at 100 Hz, and notch filtered at 60 Hz. Signals were subsequently re-referenced to the common average during preprocessing to reduce reference-dependent effects as recommended (Hernandez-Pavon et al., 2023). Ocular and muscle artifacts were corrected using independent component analysis (ICA); artifact components were identified using scalp topographies and time courses and removed before reconstruction. Continuous data were segmented into 2 s non-overlapping epochs. Epochs exceeding a 10μ*V*/*ms* gradient or 100μ*V* peak-to-peak amplitude were rejected, and bad channels were interpolated with spherical splines. Cleaned data were then spatially localized via SPECTRE.

### 2.5 Spatially resolved EEG with SPECTRE

EEG has long been considered incapable of resolving deep brain activity because of “volume conduction.” However, this limitation arises from oversimplified models that assume quasi-static fields in homogeneous tissue (Nunez et al., 1997). Our recent theoretical work shows that time-dependent electric fields in anisotropic, inhomogeneous tissue give rise to weakly evanescent transverse cortical waves (WETCOW), which provide a physical basis for reconstructing whole-brain electrical activity from surface EEG (Galinsky and Frank, 2020b,a, 2021). This framework unifies diverse spatiotemporal electrical phenomena and forms the theoretical foundation of SPECTRE (Frank et al., 2025).

**Figure 1:**
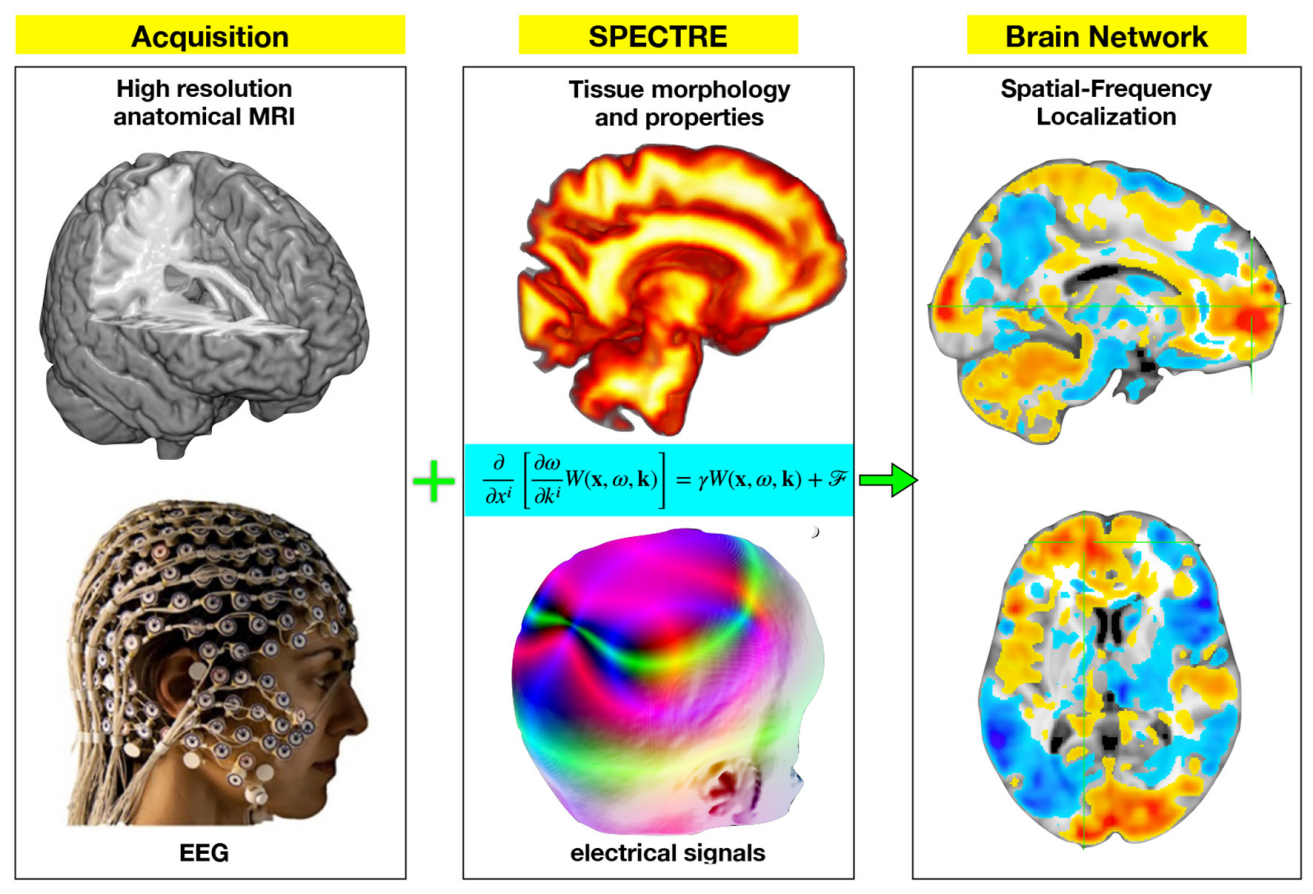
Schematics of SPECTRE framework for spatially resolving frequency-dependent EEG signals using a tissue model derived from high resolution anatomical (HRA) MRI data.

SPECTRE links scalp EEG to volumetric brain fields by combining the WETCOW forward model with high-resolution anatomical information from MRI (or a standard atlas such as MNI Fonov et al., 2009). The model accounts for brain geometry, tissue anisotropy, and inhomogeneity, providing a physically grounded mapping between recorded signals and underlying fields (Figure 1). Within this framework, Bayesian inference is used to estimate the most probable spatiotemporal configurations of the brain's electric potential. The resulting space–time modes are probabilistic eigenmodes ranked by significance, making the approach well-suited to single-subject as well as group-level analysis (Frank and Galinsky, 2016a,b; Galinsky and Frank, 2017).

In practice, SPECTRE integrates three computational components: extraction of brain geometry and tissue properties, alignment of EEG and anatomical volumes, and characterization of time-dependent spatial activation patterns. Entropy Field Decomposition (EFD) provides a compact representation of spatiotemporal activity, enabling statistical comparisons across subjects and conditions. Importantly, subject-specific MRI is not required; reconstructions can be performed on a standard atlas, facilitating broader application.

Complete mathematical derivations, algorithmic details, and validation studies are presented in our publication (Frank et al., 2025). Here we provide only a concise overview, as the focus of this work is the application of SPECTRE to TMS-evoked responses.

### 2.6 Statistical analysis

In addition to the Bayesian inference inherent to the SPECTRE framework, we performed paired-sample *t*-tests to compare BEN activity between active and sham iTBS across cortical regions and canonical frequency bands (delta, theta, alpha, beta, gamma).

Given the exploratory scope and limited sample size of this pilot study, regions with absolute *t*-values greater than 1.96 were flagged as potential candidates, consistent with prior TMS–EEG studies (Godfrey et al., 2024). For control of multiple comparisons, false discovery rate (FDR) correction was applied, and both uncorrected and corrected results are reported. To aid interpretation, we provide standardized effect sizes (Cohen's *d*), indices of cross-subject consistency, and convergence with expected stimulation-related networks. This approach emphasizes estimation rather than strict significance testing, in line with recommendations for pilot studies (Lee et al., 2014).

## 3 Results

### 3.1 SPECTRE analysis

All EEG data were co-registered to the standard Montreal Neurological Institute (MNI) T1 1mm atlas (Fonov et al., 2009) using SYMREG (Galinsky and Frank, 2018). SPECTRE was then used to estimate the electric field activation modes, incorporating tissue properties derived from the MNI T1 1mm atlas. Analyses were conducted across canonical frequency bands: delta (0.5–4 Hz), theta (4–8 Hz), alpha (8–13 Hz), beta (13–30 Hz), and gamma (30–100 Hz). For each subject, ten activation modes were calculated, from which two summary measures were derived (Frank et al., 2025):

1. Weighted summary mode (wsum), which is the eigenvalue-weighted sum of all modes, providing a probability-weighted estimate of overall network activity.
2. Unweighted summary mode (usum), which sums modes without weighting, facilitating visualization of smaller networks that may otherwise be less evident.

The wsum measure was used for statistical analysis because it provides a quantitatively accurate estimate of the relative importance of brain networks, whereas usum was reserved for visualization.

The spatial resolution of our SPECTRE analysis was defined by the HRA template (1 mm isotropic voxels), and the temporal resolution was that of the raw EEG (0.2 ms). Since the SPECTRE output was reconstructed on the MNI grid, results could be readily mapped to brain regions using the Harvard–Oxford anatomical atlas (Fonov et al., 2009).

**Figure 2:**
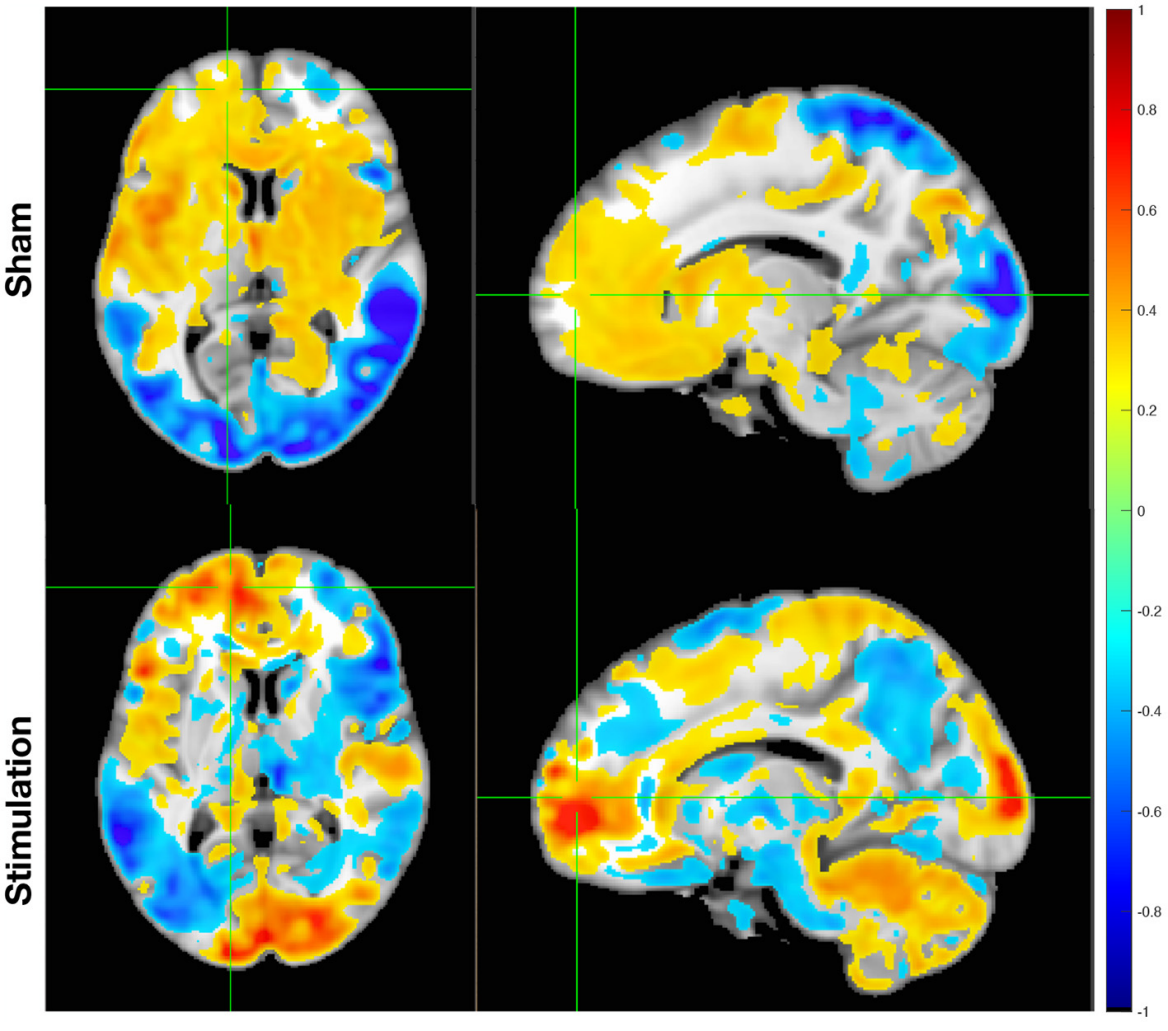
SPECTRE estimated theta band spatiotemporal electric field activation summary mode “wsum” following Sham (top) and iTBS (bottom). Activity changes during two resting-states pre- and post-stimulation are shown. Red and blue colors represent positive and negative changes, respectively. Modes have been reconstructed at 1mm spatial resolution using the MNI152 T1 weighted 1mm resolution HRA (shown as the underlaying volume with a gray-scale colormap) to derived tissue properties.

To illustrate this process, Figure 2 shows an example theta-band activation histogram from one subject under stimulation and sham conditions. The spatially and frequency-resolved differences are visually evident, highlighting SPECTRE's capacity to detect stimulation-induced changes across the entire brain, including deep subcortical regions.

An example of the average activation histogram for the wsum summary map, ranked by activation magnitude, is shown for the theta frequency range in Figure 2 for a single subject under both stimulation and sham conditions. The spatially and frequency-resolved differences between TMS stimulation and sham conditions are clearly evident, demonstrating SPECTRE's ability to quantify high-resolution electric field network activity across the entire brain, including deep subcortical structures.

### 3.2 Theta-band effects dominate post-iTBS activity

**Figure 3:**
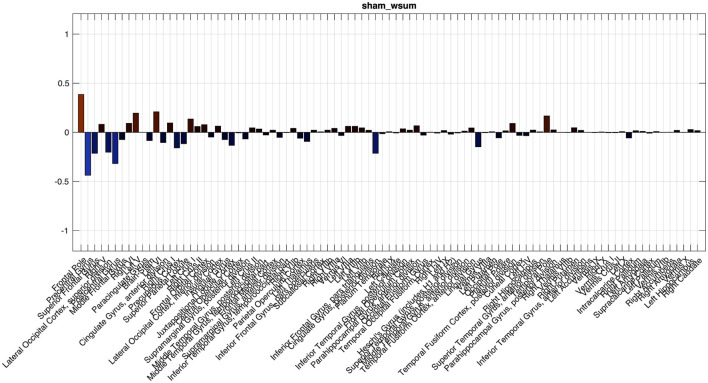
SPECTRE estimated theta band spatiotemporal electric field activation summary mode histogram for Harvard-Oxford atlas regions for Sham **(Top)** and iTBS stimulation **(Bottom)**. Both stimulation and sham histogram are sorted in the same decreasing order of activation level in the stimulation condition (bottom).

SPECTRE-based analysis of EEG revealed widespread increases in theta-band BEN activity following active iTBS compared to sham. As shown in Figure 3, theta activity differences were evident across multiple cortical and subcortical regions. For visualization, results are presented as percent change relative to sham, mapped onto the Harvard–Oxford atlas.

**Figure 4:**
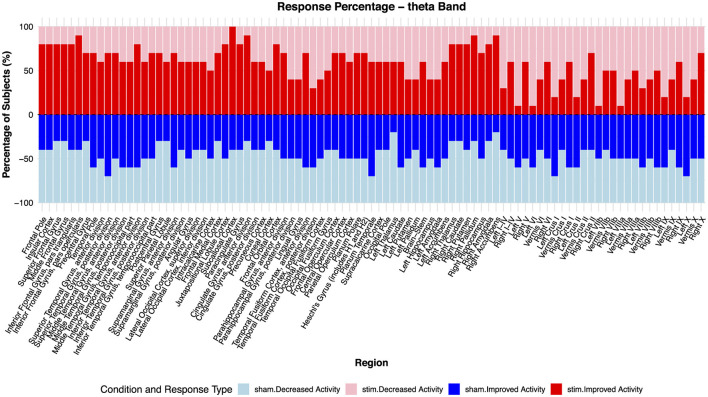
Response direction of theta frequency band across brain regions for Sham and Active TMS conditions. This figure illustrates the percentage of subjects exhibiting increased (red) or decreased (blue) oscillatory activity across different brain regions in response to sham **(Bottom)** and active **(Top)** TMS stimulation. Bars indicate the proportion of subjects showing positive or negative changes in oscillation within each brain region. The sham and active conditions are plotted separately but are sorted in the same decreasing order based on activation levels in the stimulation condition. This visualization provides insight into regional variability in response patterns across the theta frequency band.

At the individual level, responses were highly consistent: over 90% of participants showed increased theta activity in the subcallosal cortex, anterior cingulate gyrus, inferior frontal gyrus (pars opercularis), right nucleus accumbens, and right pallidum (Figure 4). Conversely, decreased theta activity was observed in cerebellar regions (left lobules V, VI, VIIB, and VIIIA) in the majority of participants. Sham sessions produced less consistent effects, with no region altered in more than 80% of participants.

**Figure 5:**
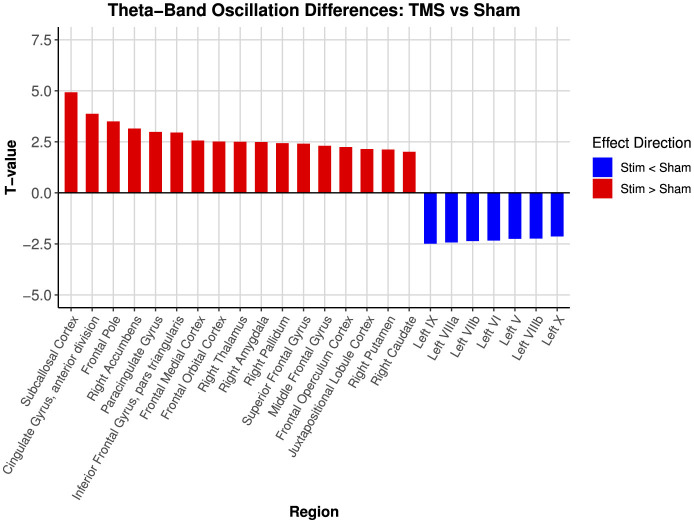
Paired *t*-test for theta-band oscillation changes in Harvard-Oxford Atlas Regions (Sham vs. Active TMS). Bar plot displaying paired *t*-test (sham vs. active) results for theta-band oscillation changes across Harvard-Oxford atlas regions following TMS. Regions are sorted in decreasing order of *t*-value magnitude. Positive *t*-values (red) indicate increased theta oscillation following stimulation (Stim > Sham), while negative t-values (blue) indicate decreased oscillation (Stim < Sham). Regions with |*T*|>1.96, corresponding to an uncorrected two-tailed *p* < 0.05, were highlighted for visualization.

Paired-sample *t*-tests confirmed these effects (Table 2; Figure 5). Significant theta increases were observed in frontal regions (frontal pole, middle frontal gyrus, superior frontal cortex), limbic regions (right amygdala, cingulate cortex), and subcortical structures (right nucleus accumbens, putamen, thalamus). In total, 17 of 91 regions (18.68%) showed significant post-iTBS increases in the theta band. Other frequency bands exhibited minimal changes: delta (0 regions), alpha (1 region; 1.10%), beta (7 regions; 7.69%), and gamma (1 region; 1.10%). Complete statistical outputs and percent-change metrics for other frequency bands are reported in Supplementary material.

**Table 2:** Paired *t*-test results for theta oscillation changes (Sham vs. Active TMS).

**Region**	***t***	***p*_*raw*_**	***p*_*fdr*_**	**Cohen's *d***
**Increased theta oscillation**				
Subcallosal cortex	4.93	0.001	0.074	1.559
Cingulate gyrus, anterior division	3.87	0.004	0.172	1.224
Frontal pole	3.50	0.007	0.203	1.108
Right accumbens	3.15	0.012	0.237	0.997
Paracingulate gyrus	2.99	0.015	0.237	0.944
Inferior frontal gyrus, pars triangularis	2.95	0.016	0.237	0.934
Frontal medial cortex	2.56	0.031	0.237	0.810
Frontal orbital cortex	2.52	0.033	0.237	0.796
Right thalamus	2.50	0.034	0.237	0.790
Right amygdala	2.49	0.034	0.237	0.788
Right pallidum	2.43	0.038	0.237	0.770
Superior frontal gyrus	2.41	0.039	0.237	0.763
Middle frontal gyrus	2.30	0.047	0.237	0.729
Frontal operculum cortex	2.24	0.051	0.237	0.710
Juxtapositional lobule cortex	2.15	0.060	0.248	0.679
Right putamen	2.12	0.063	0.248	0.671
Right caudate	2.01	0.075	0.286	0.636
**Decreased theta oscillation**				
Left IX	−2.49	0.034	0.237	−0.788
Left VIIIa	−2.43	0.038	0.237	−0.767
Left VIIb	−2.36	0.043	0.237	−0.746
Left VI	−2.33	0.044	0.237	−0.738
Left V	−2.25	0.051	0.237	−0.712
Left VIIIb	−2.24	0.052	0.237	−0.707
Left X	−2.13	0.062	0.248	−0.675

This table displays the results of paired-samples t-tests (*n* = 10) comparing mean theta power between active and sham TMS conditions across various brain regions. For each region, the table lists the *t*-statistic, Cohen's *d* effect size, the uncorrected *p*-value, and the False Discovery Rate (FDR) adjusted *p*-value (*p*_FDR_). Regions are grouped by whether theta oscillation increased or decreased during active TMS relative to the sham condition.

Effect-size analyses revealed a clear pattern: active stimulation elicited large-to-very-large increases in theta oscillations across prefrontal and limbic regions, including the Subcallosal Cortex (*d* = 1.56), Cingulate Gyrus (*d* = 1.22), and Frontal Pole (*d* = 1.11). In contrast, medium-to-large decreases were evident in cerebellar regions such as Left IX (*d* = −0.79) and Left VIIIa (*d* = −0.77). These effects did not survive FDR correction (all *p*_FDR_>0.05), but their magnitude and anatomical distribution suggest biologically meaningful changes that warrant further investigation.

Together, these results demonstrate that theta oscillations represent the most robust and consistent marker of immediate post-iTBS effects. The convergence of individual-level consistency and group-level statistics supports the utility of SPECTRE-enhanced EEG for detecting stimulation-induced network changes and highlights the theta band as a key target for future neuromodulation studies in aging and MCI.

To assess theta-band specificity within a conventional sensor-level pipeline, we conducted a *post-hoc* EEG analysis restricted to the theta band. In contrast to the robust effects observed with SPECTRE, this analysis revealed no statistically significant differences. A paired-sample *t*-tests on within-visit change scores (post–pre) showed no reliable change in global theta power between active and sham stimulation, *t*(9) = 0.70, *p* = 0.50, Cohen's *d* = 0.22. Similarly, the proportion of participants exhibiting a change in power greater than 10% was identical across conditions (90%), with 40% showing an increase in the active condition and 30% in sham.

Importantly, given the limited sample size and the well-documented low signal-to-noise ratio of scalp EEG—especially for distributed and deep network effects in TMS–EEG—the present study is not positioned to formally quantify sensor–source correspondence. Instead, the observed discrepancy underscores the limitations of sensor-level pipelines and motivates the use of spatially resolved approaches for detecting stimulation-induced network changes.

### 3.3 Limitations of the study

Given the exploratory scope and limited sample size of this pilot study, regions with absolute *t*-values greater than 1.96 were flagged as showing signals of change at a descriptive threshold. Our approach emphasizes estimation rather than strict significance testing, in line with recommendations for pilot studies. These decisions are supported by prior methodological guidance and comparable reporting practices in preliminary clinical studies (Godfrey et al., 2024; Lee et al., 2014). We note that these effects did not survive FDR correction (all *p*_FDR_>0.05), but their magnitude and anatomical distribution suggest biologically meaningful changes that warrant further investigation.

The present analysis relies on the technical validation of the SPECTRE framework described in Frank et al. (2025), and we acknowledge that full assessment of the underlying inverse solution methodology depends on that prior work. Unlike conventional EEG source localization approaches, SPECTRE computes a whole-brain inverse solution of the electric field potential without invoking discrete neuronal “sources.” In the current study, this inverse solution was implemented using a standard anatomical atlas rather than subject-specific MRI-derived head models, which may limit anatomical specificity and reduce absolute spatial precision at the individual level. This choice reflects practical constraints and an intentional emphasis on feasibility and scalability. Notwithstanding these limitations, the resulting whole-brain field reconstructions exhibit internally consistent structure and align with our a priori hypotheses, indicating that the method captures meaningful neurophysiological organization at the population level. These findings therefore establish proof-of-concept and motivate future work incorporating subject-specific anatomy and expanded validation to further refine spatial fidelity and individual-level inference.

## 4 Discussion and conclusion

The goal of this pilot study was to demonstrate the potential utility of our recently developed SPECTRE method for spatially resolved EEG in a clinically significant application–the use of TMS to mitigate memory deficits in MCI, a precursor to AD. The primary technical objective was to identify and characterize variations in BENs induced by TMS, thereby providing reproducible, quantitative metrics for monitoring and optimizing personalized treatments at the individual level. Finding should be interpreted as hypothesis-generating due to the small sample size.

Because SPECTRE is a reconstruction method that operates on standard EEG data, we were able to leverage previously acquired data from an ongoing study, enabling a retrospective application of this novel approach. Applying SPECTRE analysis to EEG data collected across multiple sessions of iTBS, we confirmed that iTBS effectively modulates BENs in older adults, with the most pronounced and consistent effects in the theta frequency band. Theta oscillations, which are critical for long-range communication and cognitive integration, showed increased activity in the limbic system (right amygdala and cingulate cortex), subcortical structures (nucleus accumbens, putamen, thalamus), and frontal regions. In contrast, several cerebellar regions exhibited reduced theta activity despite cortical enhancement, while showing increased activation in other frequency bands, a divergent pattern that may reflect adaptive redistribution of neural resources and coordinated modulation between cortical and cerebellar systems. These regional patterns should be interpreted as putative network engagement consistent with prior literature, rather than as definitive evidence of specific circuit-level mechanisms. Notably, the ability of SPECTRE to detect cerebellar modulation highlights an advantage over fMRI, which is often unreliable in these regions due to susceptibility artifacts. This observation is based on known susceptibility-related limitations of fMRI in cerebellar regions, rather than on a direct comparison performed in the present study. However, further validation, ideally through simultaneous EEG–fMRI or intracranial EEG, is necessary to fully establish the correspondence between reconstructed network-level signals and underlying neural activity.

The parietal cortex is a central node within large-scale cognitive and memory networks and maintains dense structural and functional connections with frontal, limbic, and subcortical regions. Modulation of parietal regions is therefore expected to influence distributed networks rather than produce strictly local effects. Theta-band oscillations are thought to support long-range communication across these cortico–subcortical circuits, providing a plausible mechanism by which parietal iTBS may give rise to coordinated theta changes in frontal, limbic, and subcortical regions.

Because regions were identified based on observed effect patterns, the present analyses are exploratory and susceptible to circularity; future studies should incorporate independent, hypothesis-driven ROI analyses in larger samples.

In contrast, the lack of consistent effects in other frequency bands underscores the specificity of the theta-band findings. Although there is growing interest in gamma-band activity (30–100 Hz), its reliable detection in scalp EEG remains notoriously difficult due to susceptibility to noise and muscle artifacts. Recent evidence suggests that the SPECTRE approach may help mitigate some of these limitations by improving source estimation (Frank et al., 2025), including signals from deeper brain structures that often contribute to high-frequency activity. Nevertheless, the absence of robust gamma effects in our dataset is most likely attributable to the inherently low signal-to-noise ratio of this frequency range in scalp EEG, a limitation that persists despite methodological advances. Moreover, the discrepancies observed at the sensor level highlight the inherent constraints of scalp-level EEG, where volume conduction and poor signal-to-noise can obscure subtle neurophysiological responses. Taken together, the contrast between these null results and the effects detected using SPECTRE highlights the superior sensitivity and specificity of this method for capturing the neural impact of TMS.

SPECTRE's ability to localize activation modes within specific brain regions is crucial for elucidating the neural circuits targeted in this study. Our results showed that iTBS produced consistent increases in theta activity across frontal, limbic, and subcortical regions. In particular, enhanced activity in the amygdala and cingulate cortex supports the hypothesis that stimulation engages frontal–limbic circuits central to emotional regulation and memory encoding. Complementary increases in the nucleus accumbens, putamen, and thalamus suggest modulation of cortico-subcortical loops essential for executive and motor function. The engagement of frontal–limbic and cortico–subcortical circuits suggests modulation of distributed networks rather than localized stimulation effects. Together, these findings indicate that frontal theta enhancement represents a marker of compensatory neuroplasticity, whereby neural networks adapt to support cognition in older adults. By demonstrating that iTBS can induce measurable, system-level neural changes, this work highlights the potential of TMS to engage compensatory mechanisms in aging and MCI.

SPECTRE's ability to characterize single-subject, single-study immediate post-stimulation activity in BENs following TMS enhances its clinical utility. However, our current measurements focused on single-day effects and immediate post-stimulation activity in older adults, which complicates comparisons with existing studies on resting-state EEG markers of AD/MCI, where increased frontal theta activity is often considered a marker of cognitive decline (Babiloni et al., 2021). Our measurements capture the immediate effects of TMS, probably mirroring mechanisms seen in cognitive and neural training, such as executive network recruitment and enhanced inter-regional communication (Reinhart and Nguyen, 2019). This may also partially explain the absence of significant alterations in posterior regions or other frequency bands in our data. Over longer timescales, decreased pre-stimulus theta oscillations and broader whole-brain changes may be expected due to neural reorganization and adaptation.

It is worth noting that in the current study all data were registered to a standard MNI template because anatomical MRI data were not available for all individuals. However, our SPECTRE pipeline can easily incorporate subject specific anatomical MRI data and DTI data, were they available, for analysis (prior to registration to a standard template for the purposes of group statistics) which may increase the individual specificity of the results.

Our results demonstrate that SPECTRE-EEG offers a unique combination of high temporal and spatial resolution compared to fMRI. Whereas fMRI relies on the indirect and slow BOLD response, reflecting vascular and metabolic processes on the order of seconds, SPECTRE reconstructs electric fields directly from EEG with millisecond precision, capturing the full spectrum of neural frequencies. This provides a more temporally accurate representation of brain activity.

Although this study focuses on TMS, the SPECTRE framework is broadly applicable across neuromodulation modalities. For example, *Transcranial Focused Ultrasound* (tFUS) has been shown to modulate BENs in neurodegenerative diseases, including AD and Parkinson's disease, as well as in mood disorders (Lord et al., 2024) and Brain-Computer Interfaces (BCI) (Kosnoff et al., 2024). Similarly, *Transcranial Direct Current Stimulation* (tDCS) influences BENs and has been investigated as a treatment for schizophrenia (Nitsche et al., 2008; Jog et al., 2023). By enabling high-resolution, whole-brain characterization of the effects of these interventions, SPECTRE provides a common analytic framework that can unify diverse neuromodulation approaches.

Spatially, fMRI resolution is limited by distortions and signal losses caused by magnetic susceptibility effects, particularly near air/tissue boundaries. In contrast, SPECTRE achieves resolution determined by high-resolution anatomical MRI (or atlas) and is free of the geometric distortions inherent to fMRI. EEG acquisition itself is unaffected by these distortions, making the spatial mapping more consistent and accurate.

Together, these properties highlight SPECTRE's potential as a next-generation tool for neuromodulation research. By enabling real-time, whole-brain mapping of TMS-evoked activity, SPECTRE provides a quantitative foundation for developing patient-specific protocols in brain disorders, including strategies to enhance compensatory networks in ADRD-related memory deficits; however, the present results should be considered preliminary and hypothesis-generating. Accordingly, the present findings do not confirm the stated hypotheses but rather generate testable hypotheses regarding the sensitivity of SPECTRE-enhanced EEG to stimulation-induced network changes. Future studies with larger, prospectively powered cohorts will be required to examine relationships between network modulation, cognitive phenotype, and stimulation parameters.

## Data Availability

The raw data supporting the conclusions of this article will be made available by the authors, without undue reservation.
