# Supplementary material for: Spatially resolved EEG reveals theta-band network modulation following iTBS in aging and mild cognitive impairment
Source: Front Hum Neurosci. 2026 Mar 26;20:1741133. doi: 10.3389/fnhum.2026.1741133 (PMC13061872; doi:10.3389/fnhum.2026.1741133)
Supplement: Supplementary file 1 [file Data_Sheet_1.pdf]

Supporting Information for

Spatially resolved EEG suggests therapeutic effects of TMS in AD/MCI

Lawrence R. Frank, Vitaly L. Galinsky, Hangbin Zhang, J. D. Hall, Mark W. Bondi, Ying-hui Chou

Lawrence R. Frank
E-mail: lfrank@ucsd.edu

This PDF file includes:

Supporting text
Figs. S1 to S10

Supporting Information Text

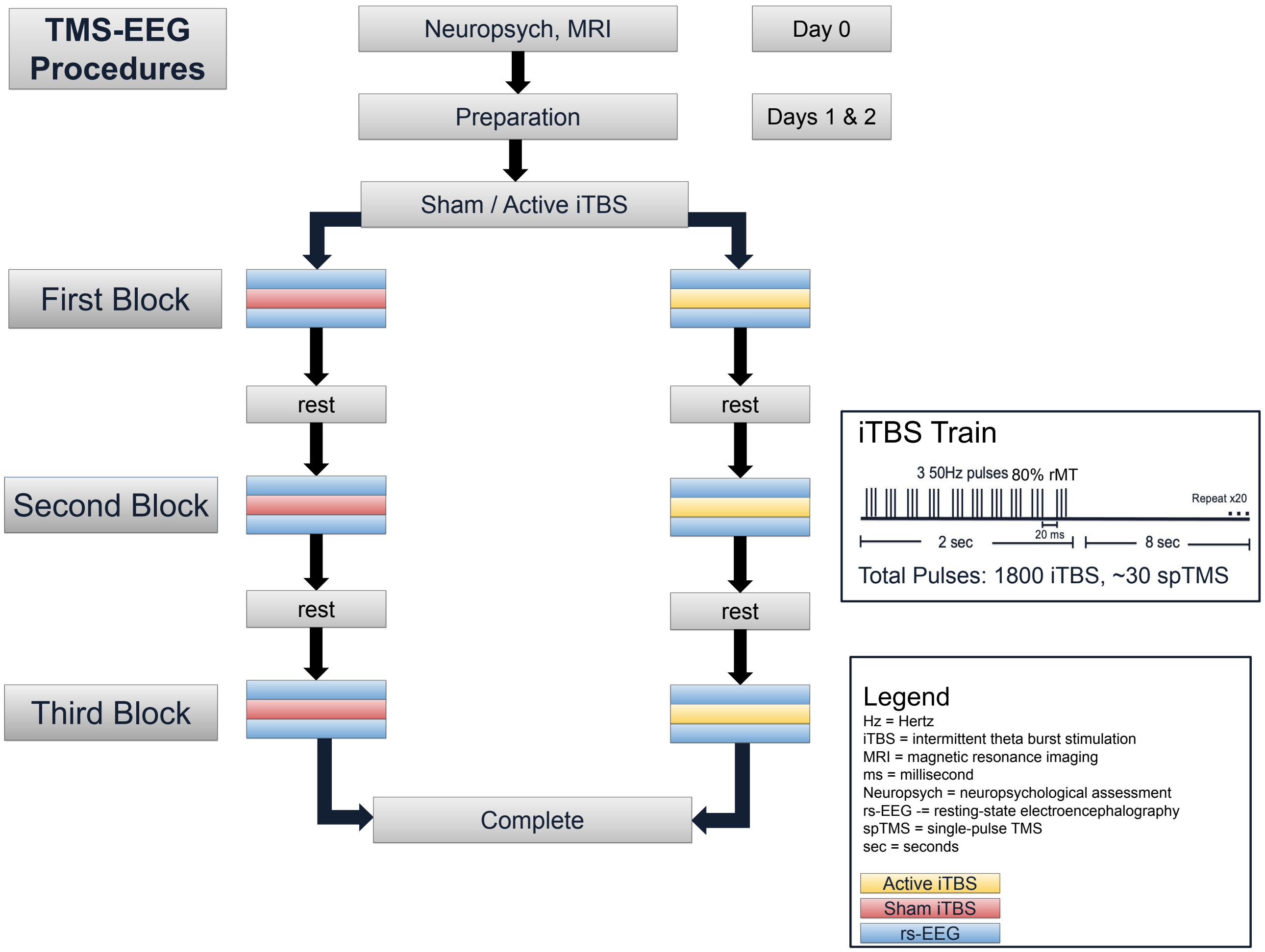

Fig. S1. TMS and EEG procedures.

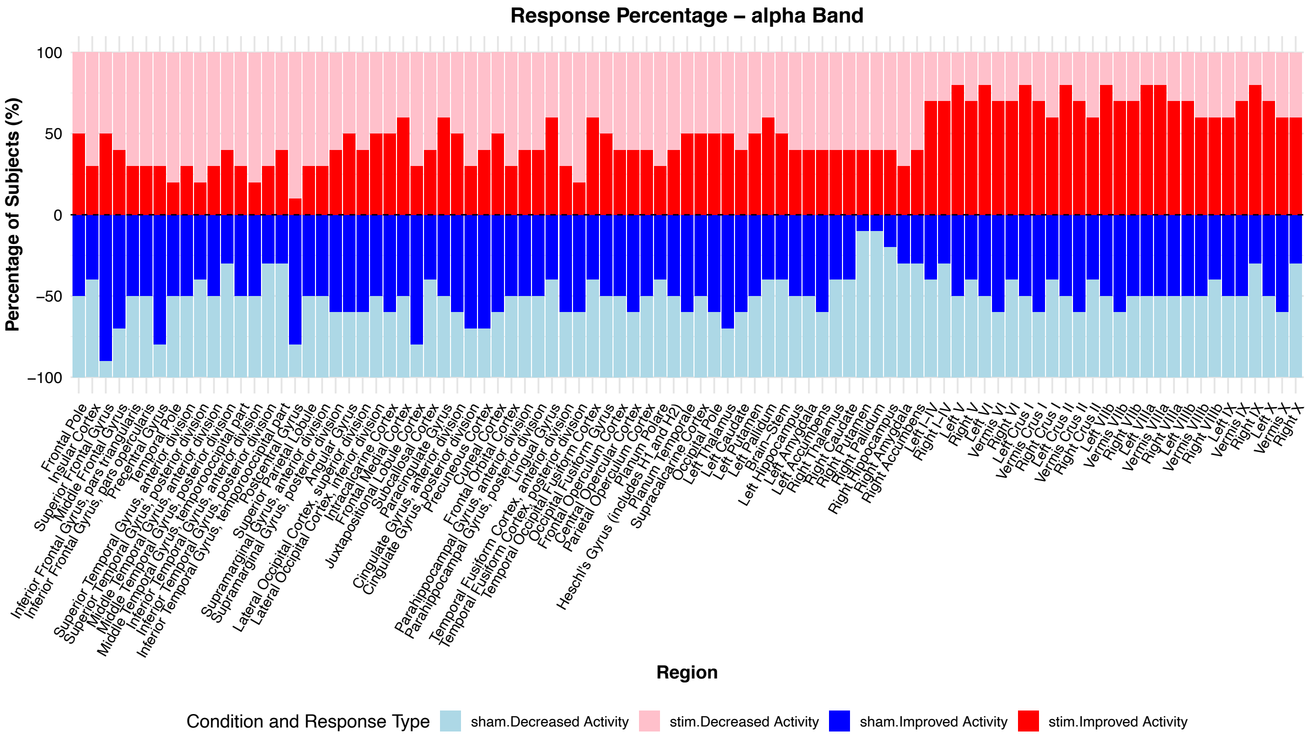

Fig. S2. Response Direction of Frequency Bands Across Brain Regions for Sham and Active TMS Conditions. This figure illustrates the percentage of subjects exhibiting increased (red) or decreased (blue) oscillatory activity in the alpha frequency band across different brain regions in response to sham (bottom) and active (top) TMS stimulation. Bars indicate the proportion of subjects showing positive or negative changes in oscillation within each frequency band. The sham and active conditions are plotted separately but are sorted in the same decreasing order based on activation levels in the stimulation condition. This visualization provides insight into regional variability in response patterns across different frequency bands.

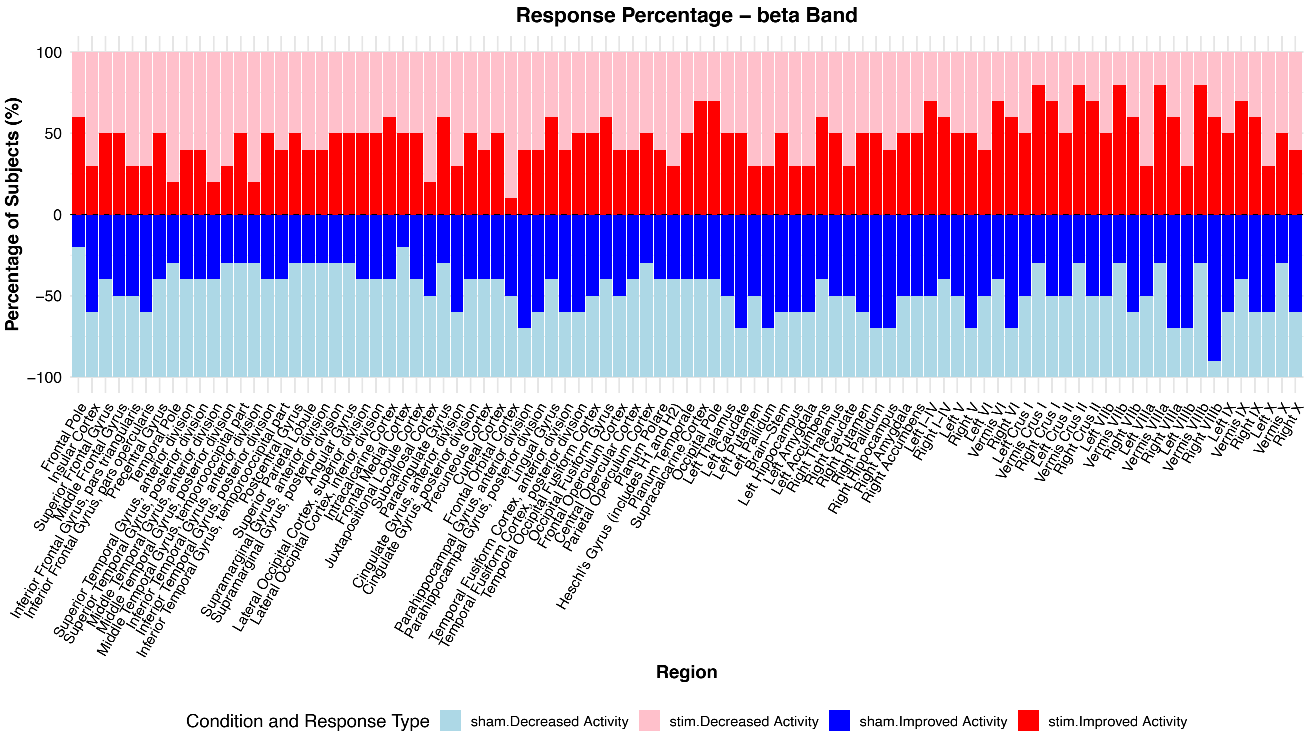

Fig. S3. Same as Figure S2 for beta band.

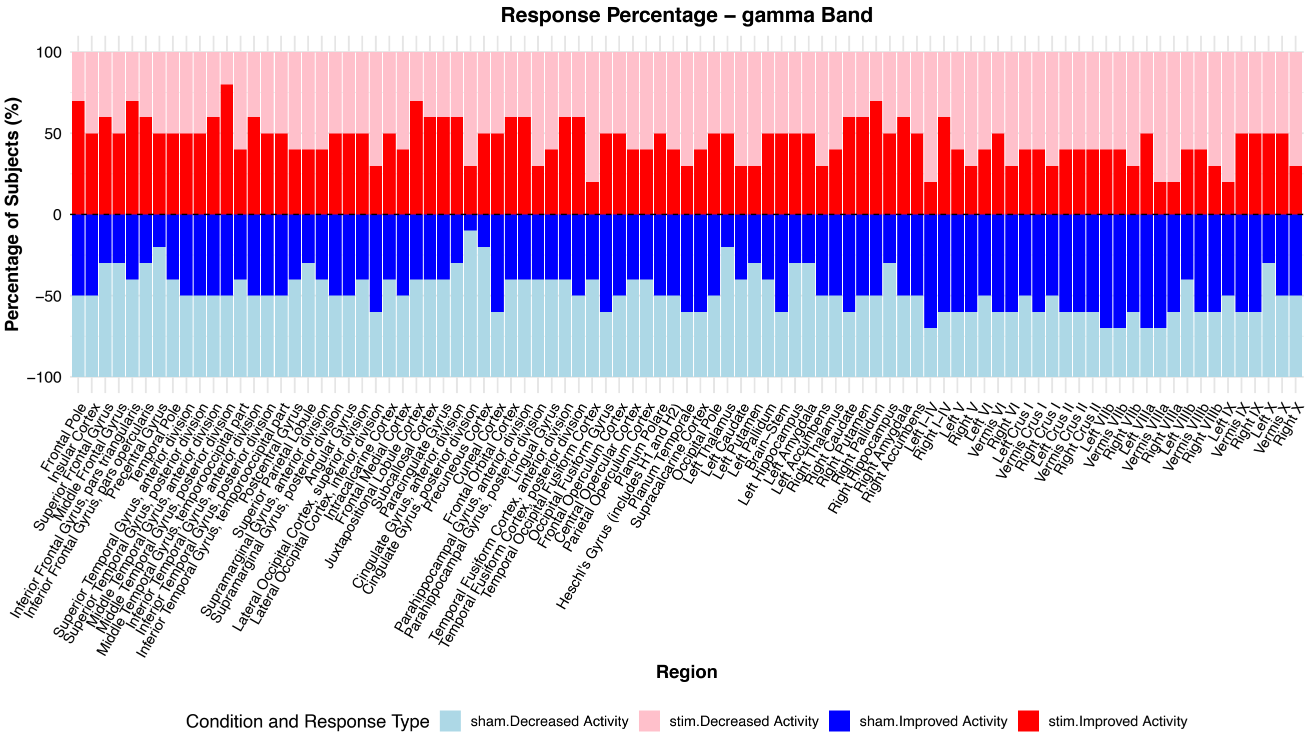

Fig. S4. Same as Figure S2 for gamma band.

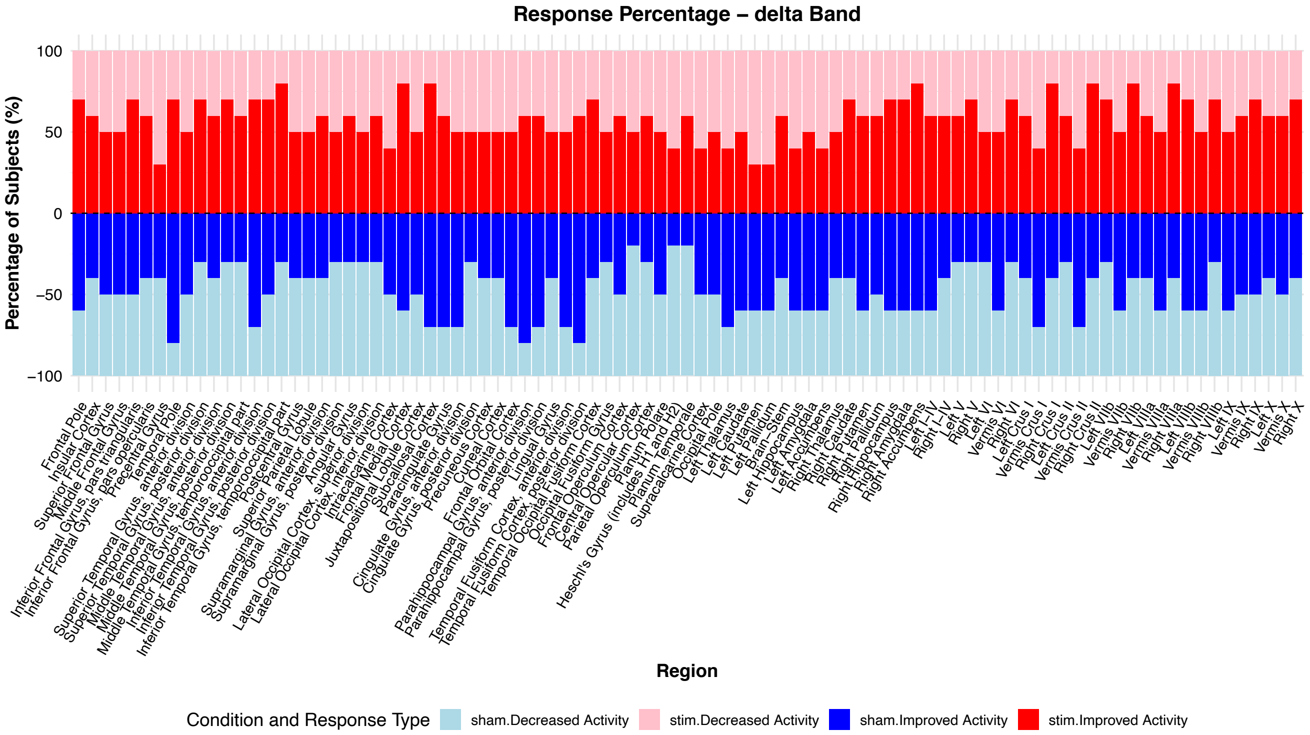

Fig. S5. Same as Figure S2 for delta band.

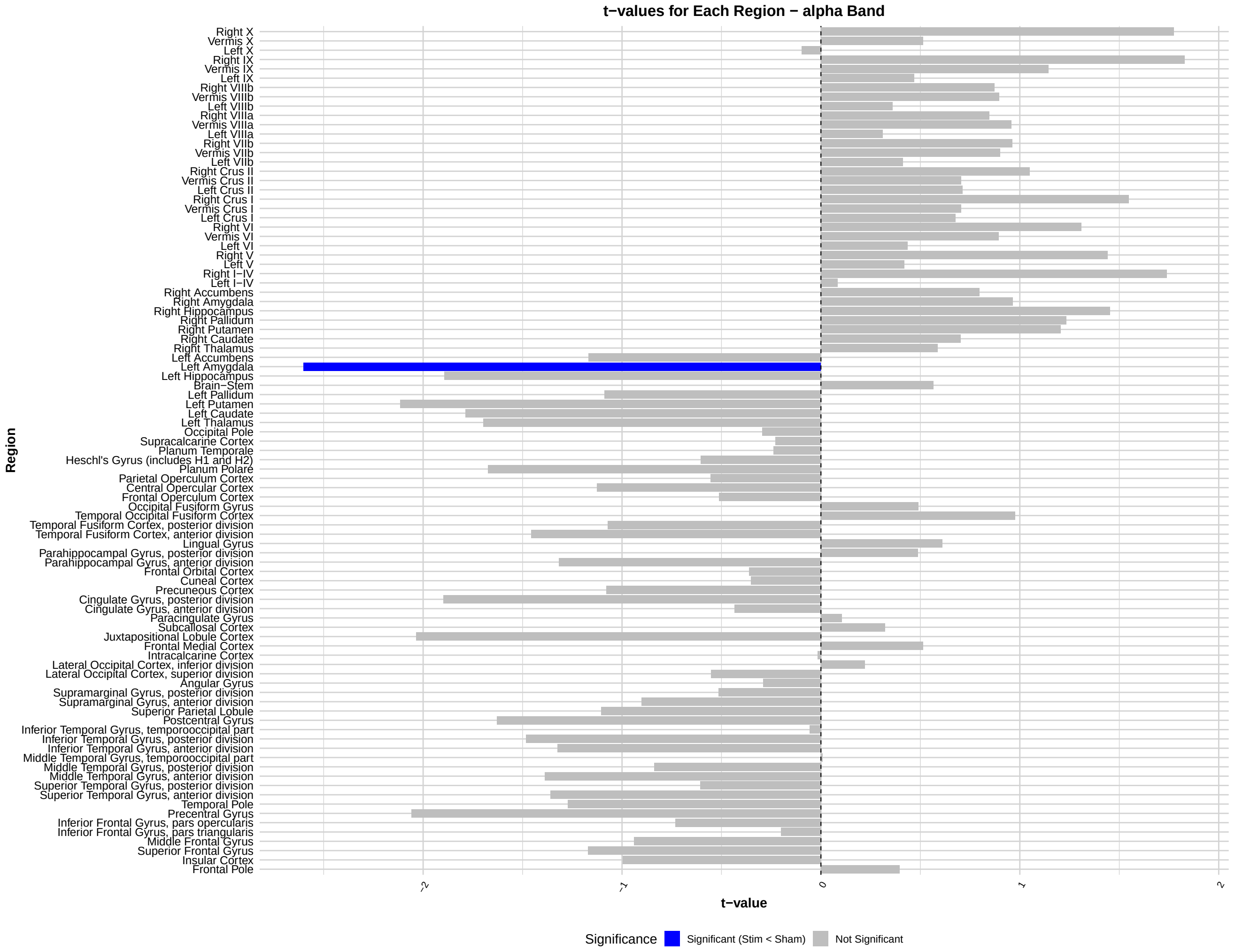

Fig. S6. t-Values for Each Region Across Frequency Bands Following Sham and Active TMS. This figure presents t-values for different brain regions across the alpha frequency band, comparing sham and active TMS conditions. Bars represent the paired t-test results, with positive t-values indicating increased oscillatory activity (Stim > Sham, red) and negative t-values indicating decreased activity (Stim < Sham, blue). Non-significant results are shown in gray.

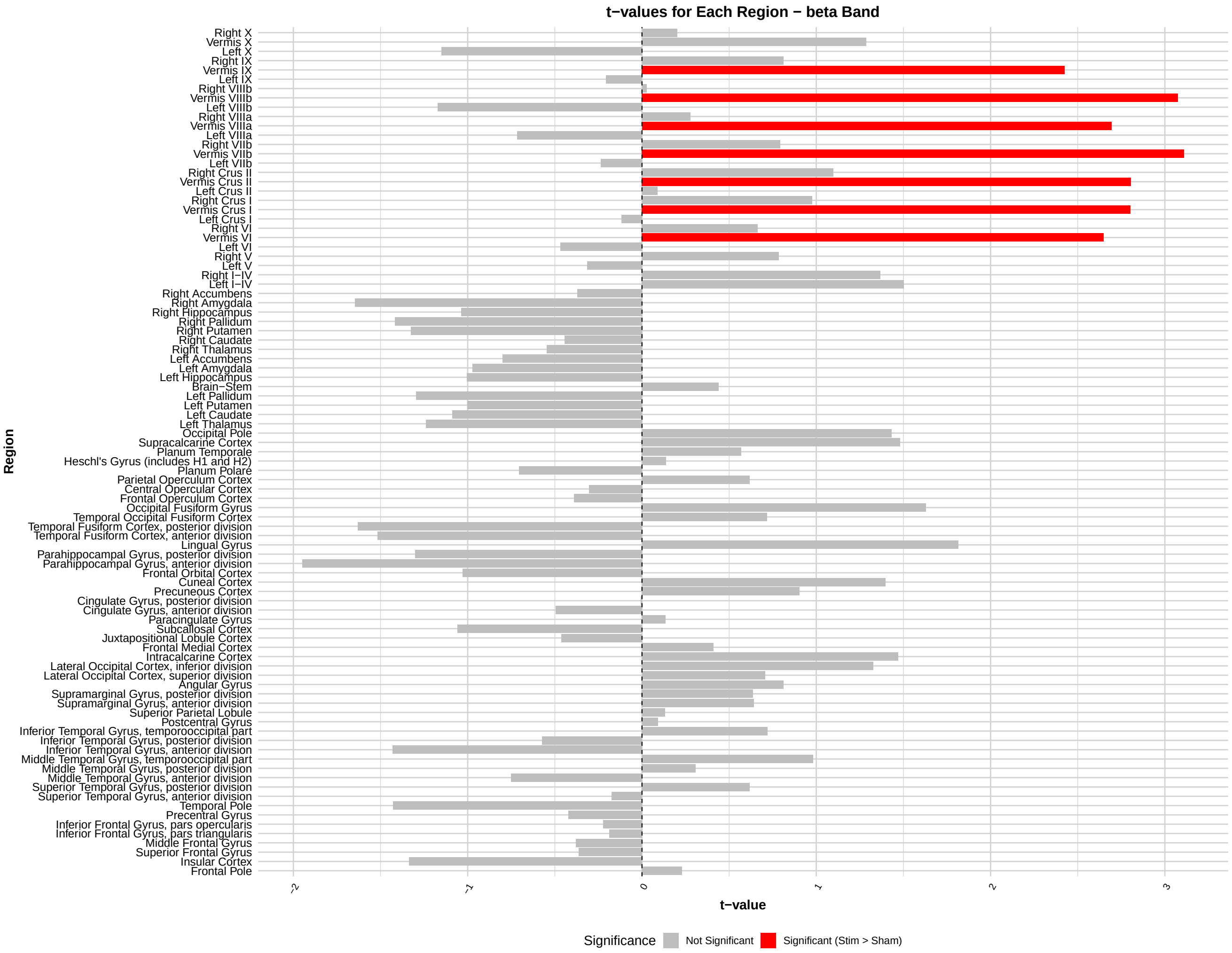

Fig. S7. Same as Figure S6 for beta band.

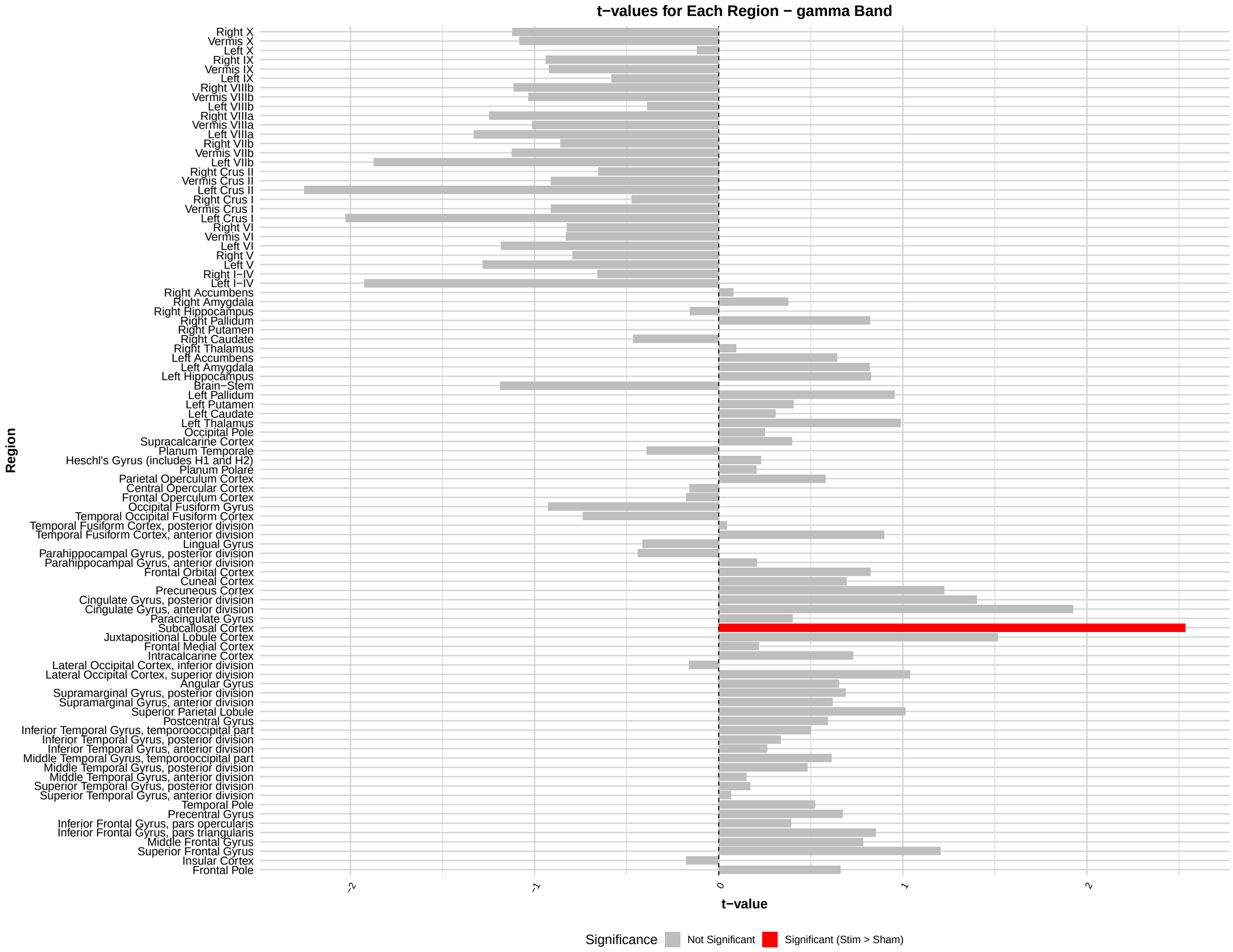

Fig. S8. Same as Figure S6 for gamma band.

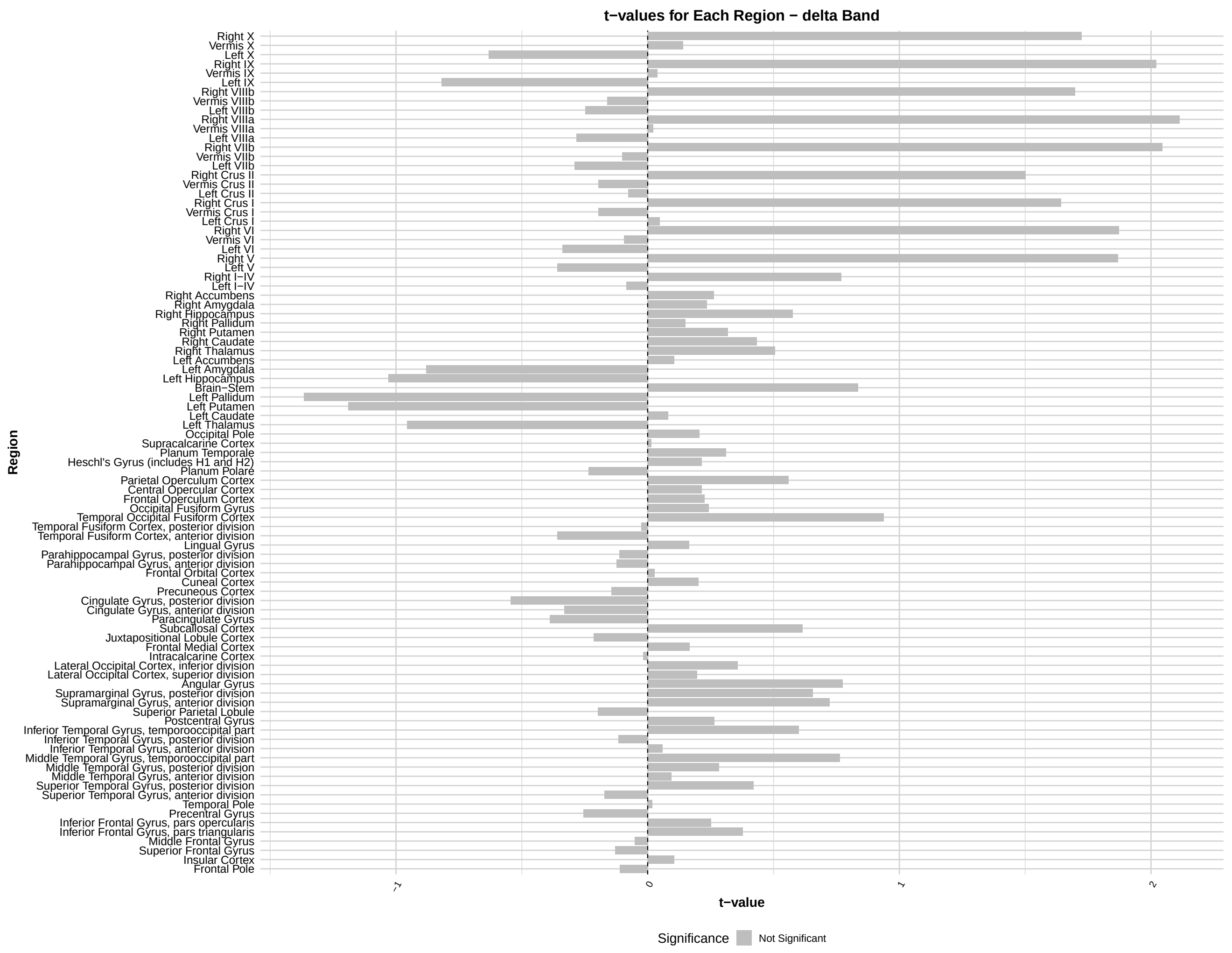

Fig. S9. Same as Figure S6 for delta band.

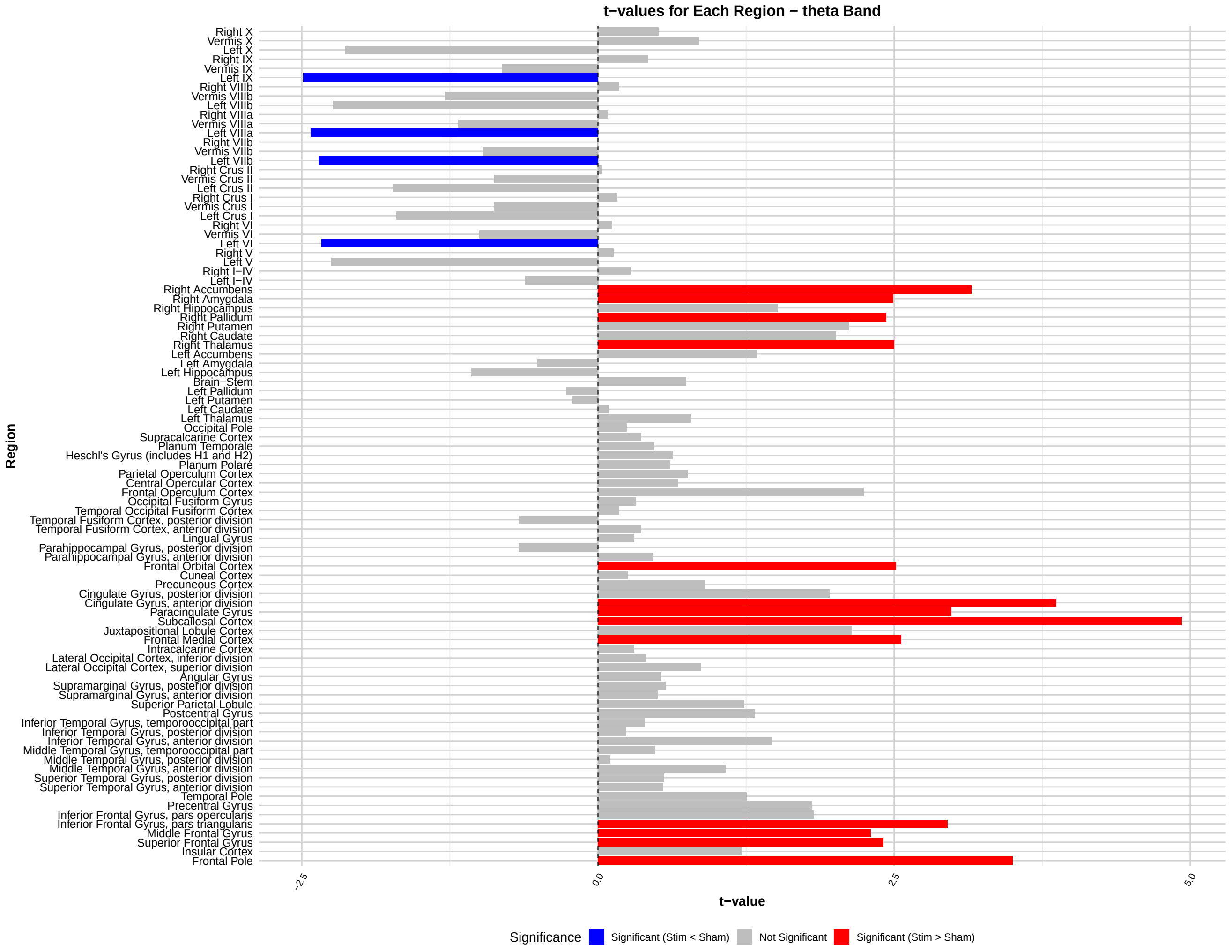

Fig. S10. Same as Figure S6 for theta band.

1. Supplementary Material: Data and Methods

Appendix 1: TMS and EEG hardware and software.

A. TMS.

A.1. Software Used.

- **Localite TMS Navigator** – Used to load MRI scans and match the orientation of the TMS coil to the hotspots for rMT and for the pre-determined stimulation sites.
- **Signal** – Employed to identify MEPs for the identification of rMT.
- **PEST** – Utilized to aid in the rMT determination process.
- **Delsys EMGWorks** – Applied for live MEP viewing and recording https://delsys.com/emgworks/.

A.2. Hardware Used.

- **Magventure TMS Coils** – MC-B70 for single pulse and Cool-B65 for iTBS https://magventure.com/products/category=coils.
- **Delsys EMG Sensors** – Two Trigno Avanti Sensors on FDI and APB muscles per subject for MEP acquisition https://delsys.com/sensors/.

A.3. Pulse Pattern(s).

- **Single pulse TMS** – Used to determine the resting motor threshold (rMT) for each subject.
- **Accelerated iTBS** – Delivered at 80% of each subject's rMT using triplets of 50 Hz pulses repeated at 5 Hz. Each session comprised twenty 2-second trains interleaved every 10 seconds, yielding a total of 600 pulses per session and 1800 pulses per day. A 6-minute rest occurred between trains.

The TMS and EEG procedures are shown schematically in Figure S1.

B. EEG.

B.1. Software Used.

- **Brainvision Recorder** https://www.brainproducts.com/solutions/recorder/.

B.2. Hardware Used.

- **Brainvision engineering actiCHamp Plus** https://brainvision.com/products/actichamp-plus/.
- **Easycap 32-channel EEG cap** https://shop.easycap.de/products/32ch-printed-empty-cap-with-acticap-snap-holders.
